# Spared Piriform Cortical Single-Unit Odor Processing and Odor Discrimination in the Tg2576 Mouse Model of Alzheimer's Disease

**DOI:** 10.1371/journal.pone.0106431

**Published:** 2014-09-02

**Authors:** Wenjin Xu, Mirielle Lopez-Guzman, Chelsea Schoen, Shane Fitzgerald, Stephanie L. Lauer, Ralph A. Nixon, Efrat Levy, Donald A. Wilson

**Affiliations:** 1 Emotional Brain Institute, Nathan S. Kline Institute for Psychiatric Research, Orangeburg, New York, United States of America; 2 Department of Child and Adolescent Psychiatry, New York University School of Medicine, New York, New York, United States of America; 3 Center for Dementia Research, Nathan S. Kline Institute for Psychiatric Research, Orangeburg, New York, United States of America; 4 Department of Psychiatry, New York University School of Medicine, New York, New York, United States of America; 5 Department of Cell Biology, New York University School of Medicine, New York, New York, United States of America; 6 Department of Biochemistry and Molecular Pharmacology, New York University School of Medicine, New York, New York, United States of America; 7 Department of Neuroscience and Physiology, New York University School of Medicine, New York, New York, United States of America; Université Lyon, France

## Abstract

Alzheimer's disease is a neurodegenerative disorder that is the most common cause of dementia in the elderly today. One of the earliest reported signs of Alzheimer's disease is olfactory dysfunction, which may manifest in a variety of ways. The present study sought to address this issue by investigating odor coding in the anterior piriform cortex, the primary cortical region involved in higher order olfactory function, and how it relates to performance on olfactory behavioral tasks. An olfactory habituation task was performed on cohorts of transgenic and age-matched wild-type mice at 3, 6 and 12 months of age. These animals were then anesthetized and acute, single-unit electrophysiology was performed in the anterior piriform cortex. In addition, in a separate group of animals, a longitudinal odor discrimination task was conducted from 3–12 months of age. Results showed that while odor habituation was impaired at all ages, Tg2576 performed comparably to age-matched wild-type mice on the olfactory discrimination task. The behavioral data mirrored intact anterior piriform cortex single-unit odor responses and receptive fields in Tg2576, which were comparable to wild-type at all age groups. The present results suggest that odor processing in the olfactory cortex and basic odor discrimination is especially robust in the face of amyloid β precursor protein (AβPP) over-expression and advancing amyloid β (Aβ) pathology. Odor identification deficits known to emerge early in Alzheimer's disease progression, therefore, may reflect impairments in linking the odor percept to associated labels in cortical regions upstream of the primary olfactory pathway, rather than in the basic odor processing itself.

## Introduction

In early stages of Alzheimer's disease (AD) and mild cognitive impairment, olfactory dysfunction has been shown to precede other cognitive impairments. Olfactory dysfunction can manifest as deficits in the detection and discrimination of odors, though in the earliest stages of AD it is most commonly expressed as an odor identification impairment [1]–[3]. Pathologically, changes typically seen in AD such as amyloid β (Aβ) accumulation and tau tangles are often detected in structures involved in olfaction such as olfactory bulb (OB) and entorhinal cortex before spreading to other areas [4]–[8]. As such, a number of olfactory assessments have been developed in the hopes that, in conjunction with other neuropsychological markers, they may serve as a method of early detection of the disease [4], [9], [10]. The effect of AD pathologies such as Aβ deposition and plaque formation and phosphorylated tau aggregation on the different olfactory processing relays in the cortex is still poorly understood. Without sufficient knowledge of how AD pathology affects the encoding and transmission of olfactory information, it is difficult to construct a complete picture of how early dysfunction in this system predicts later cognitive decline.

The piriform cortex (PCX) is the largest target of the olfactory bulb (OB) via the lateral olfactory tract (LOT) and has been shown to be intimately involved in higher order olfactory functions such as short-term odor habituation, odor discrimination and odor identification [11], [12]. While both olfactory sensory neurons [13] and the OB [14], [15] are targets of Aβ pathology, piriform cortical function is disrupted in both humans with AD [16] and animal AD models alike prior to Aβ deposition [14]. Furthermore, olfactory performance, such as odor identification in humans and habituation in mice strongly correlates with piriform cortical function in AD and AβPP transgenic models [15], [16]. For example, previous work from our group has demonstrated abnormal local field potential (LFP) hyperactivity in PCX in Tg2576 mice, which express the human amyloid β precursor protein (AβPP) with the Swedish mutation [14], similar to the hyperactivity/hyper-synchrony observed in other brain regions of AβPP mice [17]–[19]. This pathological olfactory circuit hyperactivity emerges by 6 months of age, which corresponds with deficits in olfactory habituation in early age groups [15], [20]. Furthermore, reducing soluble Aβ levels in a variety of ways restores olfactory cortical physiology and olfactory habituation [14], [20]–[22].

The present study employed the Tg2576 mouse to assess the effects of AβPP over-expression on olfactory processing in the anterior PCX (aPCX) and how this relates to behavioral performance on two olfactory tasks. The Tg2576 model over-expressing mutated hAβPP resulting in AβPP metabolite pathology is uniquely suited to assessing early, pre-depositing stages of pathology due to its relatively late onset of Aβ plaque deposition [23]. Compared to more aggressive models such as the 5XFAD [24], [25] and TgCRND8 [26], [27] which begin to exhibit plaque formation at 3 and 5 months of age respectively, the Tg2576 do not show clear amyloid plaque accumulation in cortical areas until about 10–12 months of age [23], [28]. Therefore, we are able to investigate how olfactory performance correlates with single-unit processing of odor stimuli in the aPCX of the pre-depositing brain.

Employing a combination of behavioral and electrophysiological methods, the present study found that in line with previous reports on LFP activity in aPCX [14], Tg2576 piriform cortical single-units demonstrated moderately elevated spontaneous activity. However, odor-evoked activity and odor receptive fields of these single-units were not affected by AβPP over-expression. In addition, while their olfactory habituation performance was impaired, Tg2576 olfactory discrimination in a two-odor forced choice operant discrimination task remained intact throughout age cohorts, as would be predicted given the stability of PCX odor coding. The results suggest that although AβPP metabolite pathology in Tg2576 mice can disrupt normal olfactory system excitability and simple odor memory (habituation), cortical odor coding and basic odor discrimination are robust in the face of this insult.

## Materials and Methods

### Ethics Statement

All handling, housing and experimental procedures were approved by, and performed in accordance with the Nathan Kline Institutional Animal Care and Use Committee guidelines at Nathan S. Kline Institute as well as NIH guidelines for the proper treatment of animals (IACUC protocol number AP2014-489). All efforts were made to minimize suffering.

### Subjects

A total of 36 Tg2576 (n = 12 3 months old (MO), n = 12 6MO, n = 12 12MO) and 36 B6sJLF/J wild-type (WT) (n = 12 3MO, n = 12 6MO, n = 12 12MO) littermates split evenly between male and female mice were obtained from a breeding colony at the Nathan S. Kline Institute and used in the present study for electrophysiology. Acute single-unit recordings were performed on cohorts of age matched Tg2576 and WT animals at 3, 6, and 12 months of age. In addition, a separate group of 6 Tg2576 and 6 WT, designated as an “old” (20+ MO) group were used. All animals were tested within 1 week (+/−) of the specified age ranges. A separate group of 23 WT and 6 Tg2576 mice was used to assess odor discrimination behavior in a longitudinal task spanning 10 months from 3 to 12 MO. Animals were group housed in groups of 3–4 animals per polypropylene cage until 4 days before testing, during which time they were separated and individually housed. Food and water was available *ad lib* unless noted otherwise. Lights were on a 12∶12 light:dark daily cycle, with testing occurring during the light phase. All handling, housing and experimental procedures were approved by, and performed in accordance with the Nathan Kline Institutional Animal Care and Use Committee guidelines at Nathan S. Kline Institute as well as NIH guidelines for the proper treatment of animals (IACUC protocol number AP2014-489).

### Olfactory Habituation

Animals were single-housed and kept in the vivarium prior to the olfactory habituation task to ensure that odors used in the task were encountered for the first time by test animals. The olfactory habituation task was performed as previously described in Wesson et al., 2010. Briefly, animals were kept in their home cage during testing to minimize the possible effect of novel environment on behavior. Odors were diluted 1×10^−3^ in mineral oil and applied to a cotton applicator stick which was then enclosed in a piece of odorless plastic to prevent possible contact and odor transmission to the odor port in the animal's home cage. Animals were exposed to a series of 4 different odors (heptanal, isoamyl acetate, limonene, ethyl valerate), 4 consecutive times (total of 16 exposures) with a 20 s exposure time and a 30 s inter-stimulus interval (ISI). The time animals spent investigating during odor exposure, defined as snout-oriented sniffing within 1 cm of the odor port, was measured and recorded. The 4 investigations for each odor were then normalized to the highest number of investigations for that particular odor. If an animal did not investigate a novel odor on the first presentation, all investigations for that odor were not factored into the final average.

### Odor Discrimination Task

Mice were water deprived for 23 hr/day, 5 days/week and body weights monitored to ensure no loss of greater than 10%, and most gained weight over the course of the experiment. Mice were placed in a computer controlled operant chamber (Vulintus, http://www.vulintus.com/) with 3 infrared monitored nose ports in one wall. The center port was connected to a multi-channel olfactometer (Vulintus) that delivered odorants added to a 0.5LPM clean airstream upon entry of the mouse's nose to the port. Mice were required to hold in the port for at least 300 ms before exiting to choose a reward port. Upon exiting from the odor sampling port, the mouse could poke a port to either the left or right, depending on the sampled odor identity to obtain a water reward. All trials were self initiated and most animals generated 20–100 trials in a single 20 min session. Animals were generally given two 20 min sessions/day. Initial training for all animals was with a vanilla-peppermint discrimination task. After successfully (>80% correct) mastering this task, they were switched to a mixture discrimination task. This task used odor mixtures described in Barnes et al., [29]. These mixtures were composed of 9–10 components, with concentration based on vapor pressure and dilution in mineral oil. The components used were: isoamyl acetate (100PPM), nonane (100PPM), ethyl valerate (100PPM), 5-methyl-2-hexanone(100PPM), isopropylbenzene (100PPM), 1-pentanol (100PPM), 1, 7-octadiene (400PPM), 2-heptanone (100PPM), heptanal (100PPM), and 4-methyl-3-penten-2-one (100PPM). Isoamyl acetate is removed from the 10 component mixture to create the 9 component combination and is replaced by limonene in the alternate 10 component mixture. After achieving criterion, mice were returned for 2 consecutive days of testing at 2 week intervals. Intervals for each mouse were adjusted to correspond to individual monthly and semi-monthly ages, spanning from 3 to 12 MO. The performance on the sessions within each two week time point were averaged to provide a single data point for each mouse at each time point. Performance was compared across genotype and age with repeated measures ANOVA.

### Acute Unit Recording and Odorant Stimulation

Acute single-unit recording procedures in the aPCX were performed similar to Xu and Wilson [30]. All efforts were made to ensure the health of the animals and minimize suffering. Animals were anesthetized with urethane (1.25 mg/kg) and respiration was monitored throughout the recording session with an external piezoelectric device positioned beneath the chest. Single units were recorded with a tungsten microelectrode (1–5 Mohm) and signals were acquired and analyzed with Spike2 physiology software (CED). Units were identified and separated off-line with template matching and PCA (Spike2 software) and showed at least a 2-ms refractory period in interval histograms. Layer II/III aPCX units (filtered 0.3–3 kHz) were identified with OB evoked responses, as well as histological confirmation of electrode position.

Olfactory stimuli were delivered with a flow-dilution olfactometer positioned 2 cm from the animal's nose. Odor vapor was introduced with a computer-controlled pinch valve at a rate of 0.1 liters per minute (LPM) to a constant 1 LPM flow of nitrogen gas. Stimuli were introduced for 2 s per trial with at least a 30 s inter-stimulus interval. A total of 6 odors were used (3 monomolecular, 3 odor-mixtures). Each odor was presented randomly for 4 trials for each single-unit recording. The monomolecular odorants used were ethyl valerate, isoamyl acetate and heptanal. The odor-mixtures used have been previously described [29], [31], [32] and were the same as those used in the behavioral odor discrimination task. As noted in these publications, 10C is a mixture comprised of 10 different monomolecular odors, 10C-1 is the same mixture as 10C with one component removed, and 10CR1 is the same mixture as 10C with one component replaced with a different component. The component removed in 10C-1 and replaced in 10CR1 were consistent across animals and throughout the present experiment. Both pure odorants and mixtures were diluted in mineral oil to a concentration of 100ppm based on vapor pressure. As a result, mixtures had a higher concentration than pure monomolecular odorants.

### Data Analysis

Single-unit data were all analyzed with Spike2. Single units were identified with principal components analyses as well as templating. Recordings were identified as coming from a single-unit by confirming a minimum 2 ms refractory period using interval histograms. Single-unit odor-evoked activity was defined as the spike count3 s after stimulus onset with basal firing rate (3 s pre-stimulus onset) subtracted. Odor-evoked responses were normalized to the maximal odor response (best odor) of a cell to obtain a relative response magnitude to each odor by each neuron. Spontaneous activity was defined as the per second spike rate 3 s before stimulus onset.

In addition to odor responses, single-unit entrainment to respiration was analyzed. Entrainment was calculated by filtering the respiration for 1–5 Hz frequency range and plotting a phase histogram with single-unit activity. Single-unit entrainment to respiration was performed by first extracting a time stamp for each respiration cycle. Phase plots of single-unit activity were constructed relative to these peak events and analyzed with Rayleigh statistics using MatLab sub-routines for circular statistics called CircStat [33]. MatLab was used to conduct Raleigh statistics on entrainment data. A Chi-square test was employed to check for significance between genotypes.

All statistical comparisons were done using StatView. Two-way repeated measures ANOVAs were used to compare single-unit odor-evoked receptive fields. Two-way between groups ANOVAs were used to compare spontaneous and maximal evoked single-unit activity. T-tests and post-hoc Fisher's tests were used where appropriate to make pair-wise comparisons.

### Histology and Electrode Verification

After recording, mice were sacrificed through overdosed with urethane and then transcardially perfused with PBS and 4% paraformaldehyde/PBS. Brains were removed and post-fixed in 30% sucrose/4% paraformaldehyde. Coronal brain sections (40 µm) were cut using a sliding microtome (Leica). A portion of these were mounted and stained with cresyl violet for electrode verification. The remainder sections were stored as floating sections in 0.2% sodium azide/PBS for thioflavin S staining

### Thioflavin S

Coronal sections were stained with thioflavin S as previously described in Wesson et al., 2010. Briefly, tissue samples were mounted and allowed to dry before immersion in 1% thioflavin S (Sigma-Aldrich). These were then dehydrated through immersion in increasing concentrations of ethanol before rinsing with dH2O and cover slipped.

Histological quantification for thioflavin S was performed as described in Wesson et al., 2010, using NIH ImageJ software. 4 brain areas, including OB, aPCX, hippocampus (HPX), and lateral entorhinal cortex (LEC) were analyzed. Regions of interest (ROIs) were determined using standard anatomical coordinates [34]. Images were taken at 5x magnification and thioflavin S was thresholded. Amyloid deposition was quantified as the percentage area of the total outlined area above threshold. At least 3 coronal sections were averaged per animal for each brain area. One-way ANOVAs were used to compare thioflavin S area fractions across age groups for each brain region.

### Immunohistochemistry

Coronal sections were treated with anti-Aβ antibodies (6E10). Immunohistochemistry was performed as previously described in Wesson et al. [15]. Briefly, sections were rinsed and blocked for 1 hour with 20% filtered normal goat serum diluted in PBS. Samples were then incubated in 6E10 (1∶200) overnight at 4°C. Samples were rinsed with PBS and incubated for 2 hours in Alexafluor 488 anti-mouse secondary antibody (1∶500). After incubation, tissue was rinsed a final time, mounted onto glass slides, dried and covered using GelMount. Staining groups always included sections from each age group and genotype.

Histological quantification for thioflavin S and 6E10 was performed as described in Wesson et al., 2010. Quantification of Aβ was performed with NIH ImageJ software. 4 brain areas, including OB, aPCX, HPX and lateral entorhinal cortex (LEnt), were analyzed. Regions of interest (ROIs) were determined using standard anatomical coordinates [34]. Images were taken at 5x magnification and thioflavin S and anti-Aβ florescence were thresholded. Aβ deposition was quantified as the percentage area of the total outlined area above threshold. At least 3 coronal sections were averaged per animal for each brain area. One-way ANOVAs were used to compare thioflavin S and anti-Aβ area fractions across age groups for each brain region.

### Gender effects

Previous groups have reported possible gender effects in Tg2576 wherein females accumulated Aβ pathology and expressed behavioral deficits earlier than male counterparts [26], [35]. Thus, one-way between groups ANOVAs were performed investigating this possible gender effect on the physiological, behavioral and immunohistochemical measures reported here. These revealed no significant gender differences on any measures in the present study (data not shown). As such, all final statistical comparisons made were collapsed across gender.

## Results

### Piriform cortical single-unit spontaneous activity

**Figure 1:**
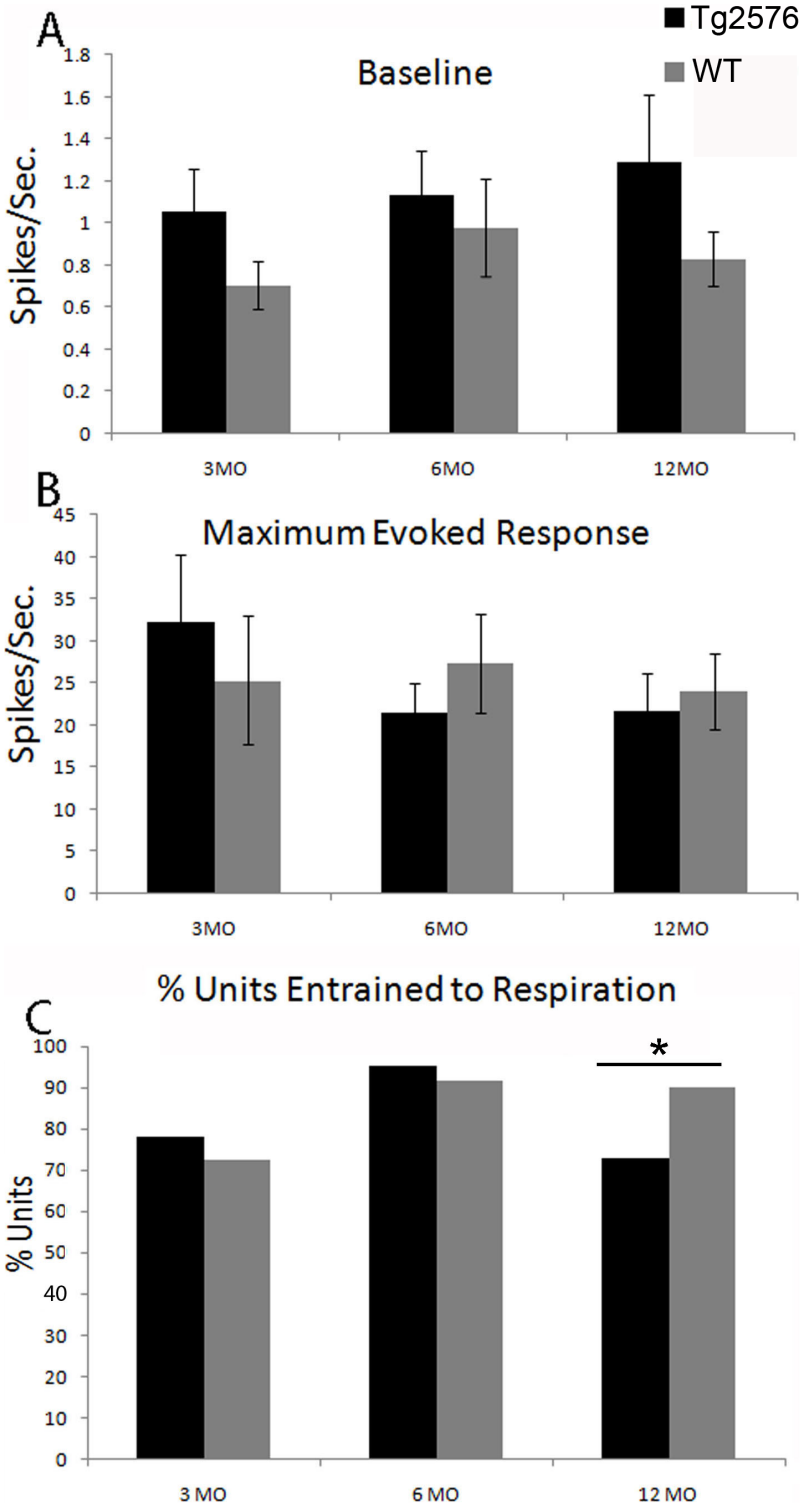
Single unit activity in Tg2576 versus age-matched WT mice at 3, 6 and 12 MO. Tg2576 showed a trend towards higher baseline activity (A) versus age-matched WT (*p* = .06) but no difference in highest odor-evoked response (B). Unit entrainment to respiration (C) was diminished in Tg2576, though this did not emerge until 12 MO (* = *p*<.05). Data presented as mean ± SEM.

A total of 110 WT (n = 29 for 3MO, n = 39 for 6MO, n = 42 for 12MO) and 110 Tg2576 (n = 35 for 3MO, n = 41 for 6MO, n = 34 for 12MO) single-units were recorded and tested for odor responses, with a mean of 3.5±0.2 single-units/animal and no more than 9 single-units acquired in any one animal. Piriform cortical single-units in Tg2576 mice showed a trend toward increased baseline firing at all ages compared to single-units in WT mice (*F*(1,207) = 3.45, *p* = 0.06) (Fig. 1A). This elevation is consistent with the heightened power of LFP oscillations previously reported [14]. In contrast to the spontaneous activity, the maximal odorant-evoked firing rate of single-units was not significantly different from WT at any age (*F*(1,207) = .564, *p* = N.S.) (Fig. 1B).

The temporal structure of single-unit activity varied with genotype. For each unit, phase locking of single-unit activity to respiration was examined with Rayleigh statistics as previously described [30]. Figure 1 shows the percentage of units with activity significantly entrained to respiration (Figure 1C). Entrainment to respiration was significantly reduced at 12 MO in Tg2576 mice compared to WT (*χ^2^* ^2^(1) = 3.91, *p*<.05), but no change was observed at earlier ages. Thus, although the effects were small, both overall single-unit baseline activity levels and the temporal structure of this activity were modified in Tg2576 mice compared to WT controls, with the temporal structural modification occurring during more advanced stages of pathology development.

### Tg2576 mice show no change in single-unit odor receptive fields in piriform cortex

**Figure 2:**
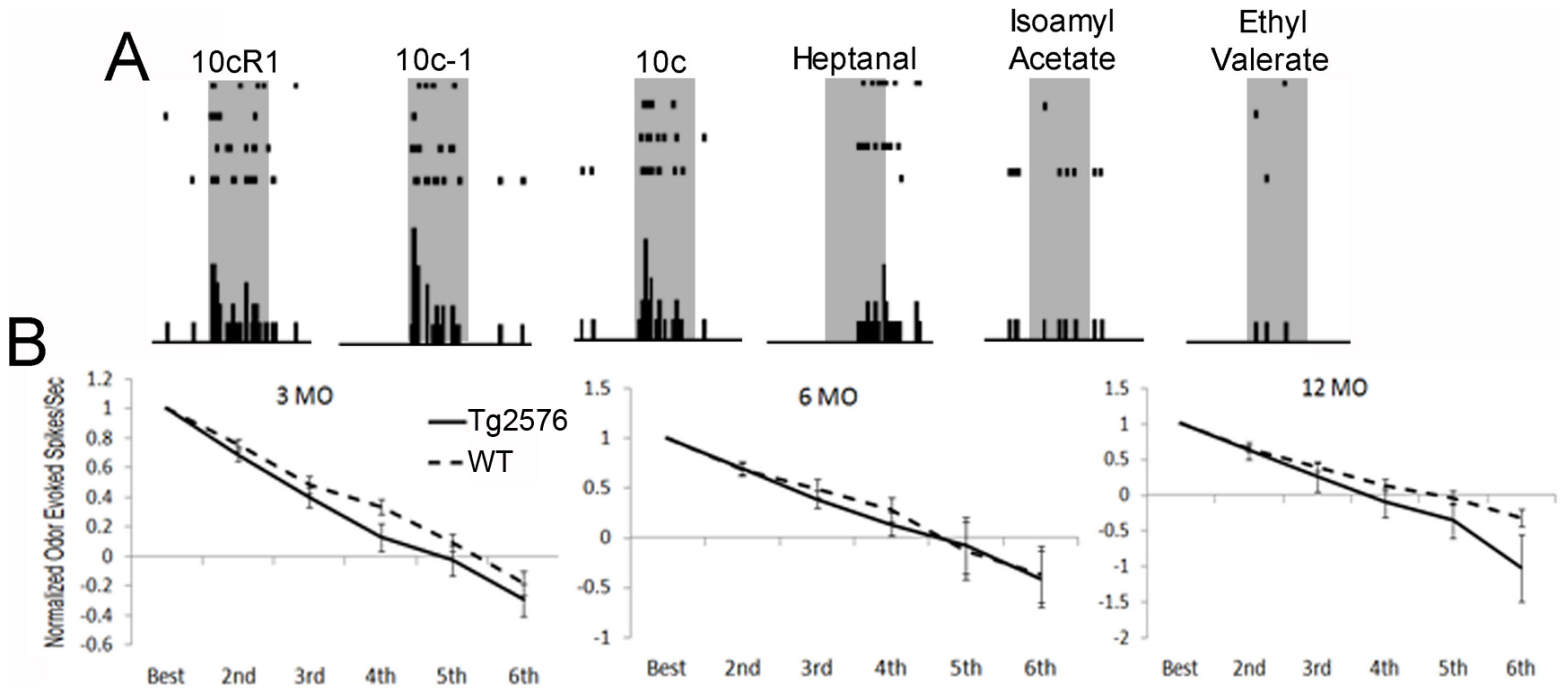
Single unit receptive fields in aPCX. (A) Representative single-unit response to stimulus set. Rasters represent unit activity from a single cell tested with multiple odors and histogram indicates tally of rasters for each odor. Shaded area indicates 2 seconds starting at the onset of stimulus delivery. (B) Odor receptive fields in Tg2576 versus age-matched WT at 3, 6 and 12 MO. X-axis is odor stimuli organized by response strength. Y-axis is odor-evoked spikes per second normalized to the highest response of the six odors. Tg2576 single-units showed no difference in receptive field specificity compared to age-matched WT mice.

Previous work has demonstrated a link between the breadth of aPCX single-unit odor receptive fields (or response range) and behavioral perceptual acuity [29], [31], [36]. Thus, as a first examination of the effects of hAβPP over-expression on aPCX odor coding precision, we examined odor receptive fields across age and genotype. After single-units were isolated, 6 different odors were administered 4 times each in random order (Fig. 2A). Receptive fields were calculated by taking the highest (mean) response rate in the 6 odor set and normalizing the other 5 odor responses to it. These normalized response magnitudes were then ordered from highest to lowest response and compared across genotypes. No difference in odor receptive fields, based on a diverse set of monomolecular and mixture stimuli, was detected (Fig. 2B). Repeated measures ANOVA revealed no significant difference between piriform cortical single-unit odor receptive fields of Tg2576 and WT mice at any age (*F*(1,207) = 1.95, *p* = N.S.). Therefore, Tg2576 showed intact single-unit odor processing that was comparable to age-matched WT mice.

### Tg2576 are impaired in simple odor memory but not in odor discrimination

**Figure 3:**
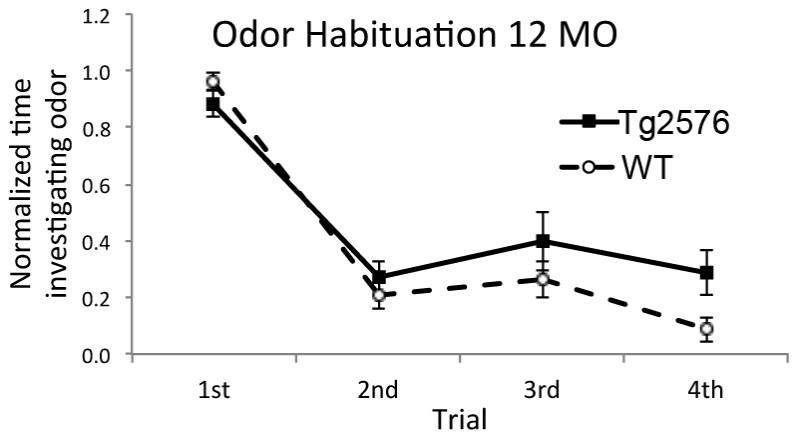
Short-term odor habituation was impaired in Tg2576 mice compared to WT controls. For example, as shown here, 12 MO Tg2576 mice (n = 12) showed less habituation over the course of four repeated odor stimuli than age-matched WT controls (n = 12).

Consistent with previous reports [15], [20], [37], [38] simple odor memory (short-term odor habituation) was impaired in Tg2576 mice compared to WT controls (genotype X age X habituation trial ANOVA, main effect of genotype, (*F*(1,192) = 5.73, *p*<0.02). Figure 3 shows habituation data from 12 MO Tg2576 and WT mice (n = 12 each). In rodents, short-term odor habituation is a behavioral read-out of piriform cortical function [39], [40], as is, in part, behavioral odor discrimination [29], [31], [41]. The single-unit sensory physiology results described above suggest that there may be intact odor discrimination in Tg2576 mice compared to WT. To examine this, animals were tested longitudinally in a two-alternative forced choice odor discrimination task using highly overlapping odorant mixtures as test stimuli. Previous work in rats has demonstrated that discrimination of overlapping odorant mixtures varies with the nature of that overlap [29], [32]. Thus, rats can relatively easily learn to discriminate a 10 component mixture (10c) from that same mixture with one component replaced with a novel contaminant (10cR1), i.e., a 90% overlap. However, it is significantly more difficult for rats to discriminate the 10 component mixture from the same mixture with one component missing (10c-1), even though that is also a 90% overlap. Successfully learning this latter, more difficult task also induces a variety of changes in piriform cortical sensory physiology [31].

**Figure 4:**
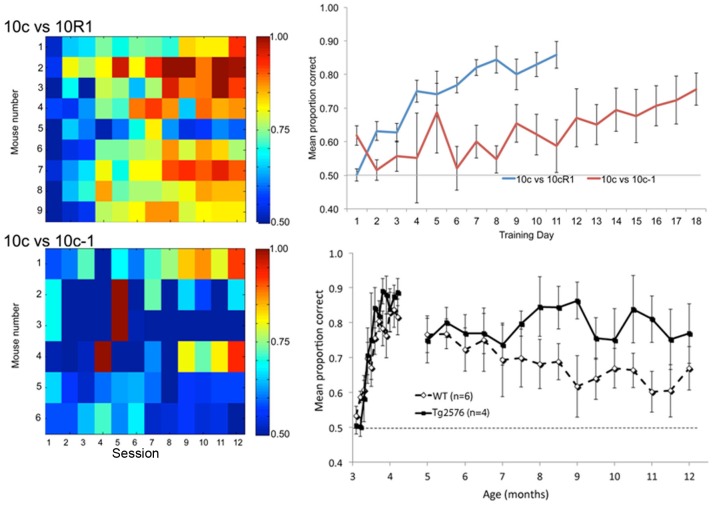
Behavioral discrimination of overlapping mixtures in WT mice. The pseudo-color panels (color corresponds to proportion correct) show performance for individual mice in each task. Mean performance for each task (top right). Different mice were used for each task. As previously shown in rats, the 10c vs. 10cR1 discrimination was significantly easier to acquire than 10c vs 10c-1. However, so few individual animals acquired the 10c vs. 10c-1 task it was not feasible to use it to test the effects of APP over-expression. There was no significant difference between WT and Tg2576 mice in performance on an odor mixture discrimination task (10c vs. 10cR1) across age (bottom right). Of the initial 7 WT and 6 Tg2576 mice, 1 WT and 2 Tg2576 animals died prior to 12 months and are not included here. Animals were trained in the two-alternative forced choice task prior to 5 months of age and then tested bimonthly until 12 months. Initial acquisition of the discrimination was not affected by genotype. Furthermore, there was no significant effect of genotype on performance of this well learned odor discrimination task through 12 months of age.

As a first step, we confirmed that odor mixture discrimination in B6sJLF/J mice was similar to that reported in rats. Adult WT mice were trained in a 2-alternative forced choice operant task for water reward to discriminate either 10c from 10cR1 (n = 9) or 10c from 10c-1 (n = 6). As reported in rats, mice learned the 10c versus 10cR1 much faster than 10c versus 10-1 (Fig. 4). However, unlike rats, the majority of mice never successfully achieved criterion (80% correct) on the difficult task. Thus, we were limited to using the simpler, 10c versus 10cR1 discrimination here.

To test the effects of AβPP over-expression on odor mixture discrimination, Tg2576 (n = 4) and WT (n = 6) mice were initially trained in the 10c versus 10cR1 task between 3–4.5 months of age. As shown in Fig. 4, animals of both genotypes rapidly learned the discrimination, with no significant genotype difference (repeated measures ANOVA over the initial training session, genotype X trial; main effect of trial, *F*(11,88) = 19.54, *p*<0.01; main effect of genotype, *F*(1,88) = 0.44, N.S.). Furthermore, there was no significant effect of AβPP over-expression on performance of this well learned odor discrimination task through 12 months of age, though Tg2576 actually had a trend toward enhanced performance (repeated measures ANOVA genotype X age, main effect of age, (*F*(14,112) = 0.66, N.S.; main effect of genotype, *F*(1,112) = 2.11, N.S.). Although counter to our original hypotheses of impaired odor perception in AβPP mice, these behavioral psychophysical results correspond well with the maintained single-unit sensory coding within the aPCX described above.

### 20+ Month old Tg2576 show no single-unit odor receptive field changes from age-matched WT mice

**Figure 5:**
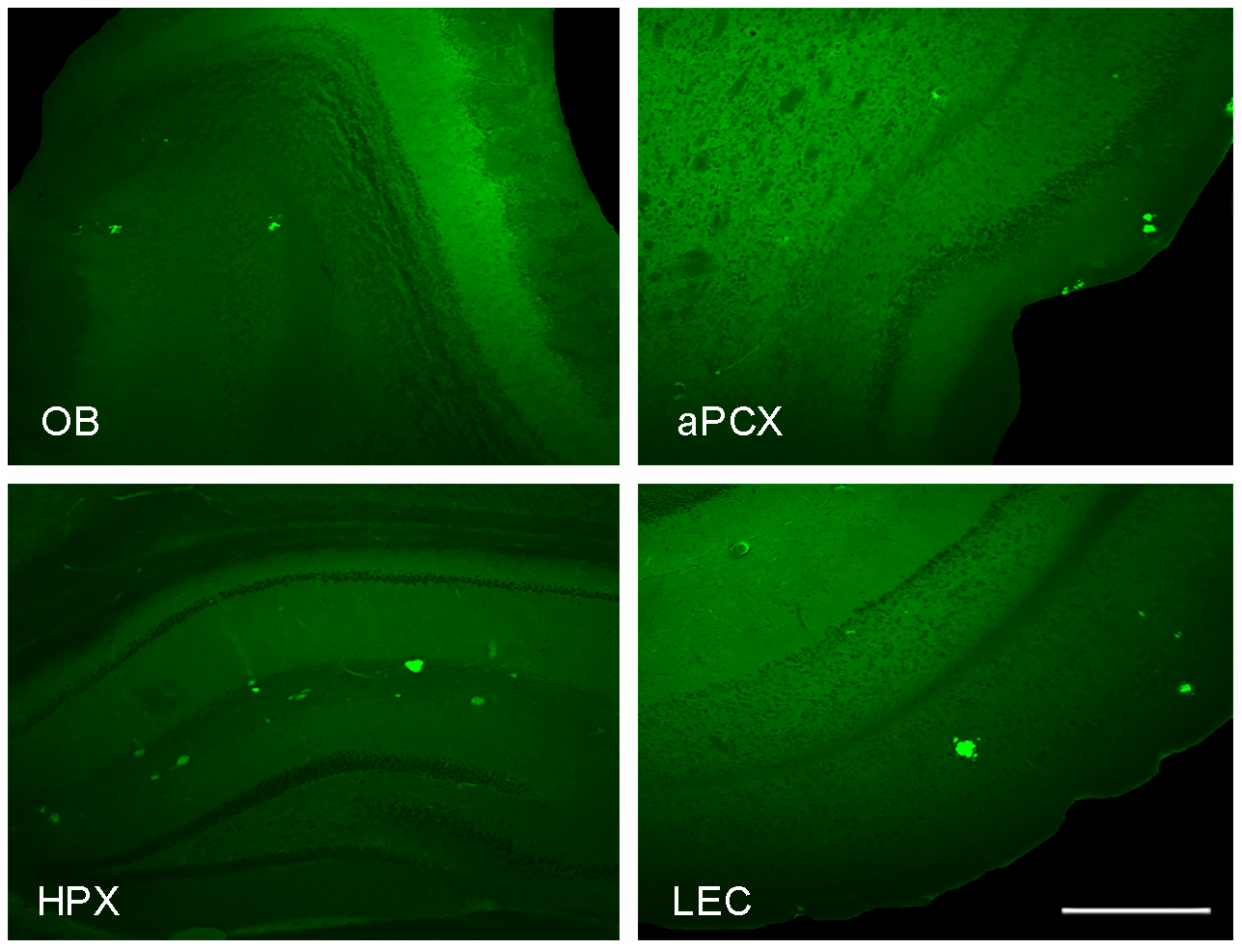
Histological examples of thioflavin S staining at 12 MO in Tg2576 mice for olfactory bulb (OB), anterior piriform cortex (aPCX), hippocampus (HPX) and lateral entorhinal cortex (LEC). Scale bar is 500 µm.

To confirm the phenotypic development of amyloid pathology in the animals in the present study, staining for plaques (thioflavin S) and immunohistochemistry for Aβ deposition (6E10) were conducted. Similar to previous observations [15], there was a significant effect of age in Tg2576 mice for thioflavin S deposition in the aPCX (Fig. 5). 12 MO animals showed greater staining than 3 and 6 MO age groups and both 3 and 6 MO age groups showed little to no thioflavin S and soluble Aβ staining.

**Figure 6:**
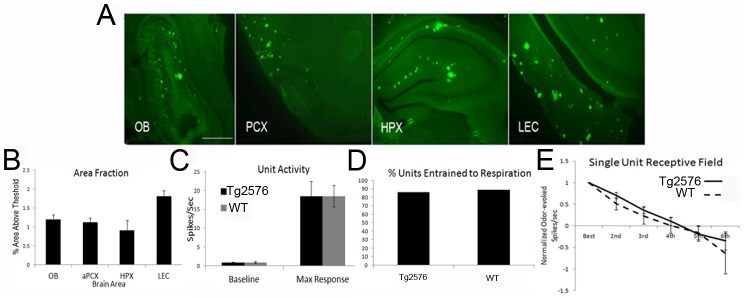
Summary of data obtained for 20+ month old Tg2576. (A) Mice over 20 months of age showed abundant thioflavin S positive staining in (from left to right) OB, anterior piriform cortex (aPCX), hippocampus (HPX) and lateral entorhinal cortex (LEC). Scale bar is 500 µm. (B) Thioflavin S area fractions for 20MO animals in OB, aPCX, HPX and LEC. (C) 20 MO Tg2576 showed no difference in baseline or maximal odor-evoked unit activity and (D) no difference in unit entrainment respiration. (E) Finally, no difference was observed in single-unit receptive field specificity compared to age-matched WT.

Given the remarkable stability of odor processing in Tg2576 mouse piriform cortical single-units during early ages, a separate group of 20+ (20–24 MO) Tg2576 and age-matched WT mice (n = 6 each) were assessed on the same battery of physiological measures to reveal the effect of late-stage amyloid pathology (with a mean of 4.6±0.5 single-units/animal and no more than 7 single-unit acquired in any one animal). By 20 MO, all olfactory cortical areas contained abundant amyloid staining (Fig. 6A, B). However, piriform cortical single-unit baseline (n = 27 units for WT, n = 28 for Tg2576, *F*(1,53) = .02, *p* = N.S.) and maximal odor-evoked (*F*(1,53) = .00, *p* = N.S.) activity in 20+ MO Tg2576 mice were not different from single-units in WT mice, nor were there changes in entrainment to respiration (*χ^2^*(1) = 0.12, *p* = N.S.) (Fig. 6C, D) or odor receptive field specificity (*F*(1,37) = .38, *p* = N.S.) (Fig. 6E). The results suggest remarkable stability of aPCX odor coding during even extreme amyloid deposition.

## Discussion

The present results suggest that in the face of advancing pathology created by amyloid β, odor coding and olfactory perceptual acuity remain surprisingly robust in an animal model of AD. These data support recent behavioral observation in other animal models of AβPP over-expression [42], [43], and for the first time demonstrate that the spared odor discrimination behavior reflects intact aPCX single-unit odor coding. Disruption in all phases of odor perception have been described in AD, including detection, discrimination and identification, however, odor identification deficits appear to be the first to emerge early in AD progression. The stability of odor discrimination and cortical odor coding observed here, therefore, may suggest that odor identification problems in AD reflect impairments in linking the odor percept to associated labels in cortical regions upstream of the primary olfactory pathway, rather than in basic odor processing itself. It must be noted that while care was taken to utilize odorant stimuli that were highly overlapping and previously demonstrated to be difficult to discriminate [29], [31], [32], [44], use of lower intensity stimuli or more highly similar stimuli may detect impairments that could be detected with the present techniques.

Over-expression of mutant hAβPP in mouse olfactory sensory neurons can impair behavioral odor discrimination [45]. However, more central deposition of Aβ has not been found to significantly impair behavioral odor discrimination in mice co-expressing the hAβPP with the Swedish mutation and human mutant presenilin-1 [42], nor in APP23 mice over-expressing the Swedish mutated hAβPP alone [43]. Here we demonstrate for the first time this lack of detectable impairment in behavioral odor discrimination in Tg2576 mice over-expressing the Swedish mutated hAβPP that corresponds well with the lack of detectable change in piriform cortical odor single-unit acuity. There are a variety of olfactory behavioral assays which vary in their specific sensitivity to different olfactory impairments. Vloeberghs et al. [43] used food finding test that required identification and localization of food pellets by smell in APP23 mice and found no olfactory deficit. Phillips et al. [42] used a Go-No-Go task with monomolecular odorants to assess olfactory thresholds and discrimination and found no deficits in either aspect in AβPP_SWE_ X PS1 mice. Here, we used a two-alternative forced choice task with overlapping mixtures to assess discrimination in Tg2576 mice and similarly found no behavioral impairment. Thus, using a variety of tasks and odors, AβPP over-expression and Aβ deposition in the olfactory system does not appear to disrupt odor discrimination. We have previously suggested that Tg2576 mice have an odor discrimination impairment when tested with a cross-habituation task [15] rather than the two-alternative forced task used here. However, interpretation of the cross-habituation data must be tempered by the fact that levels of self-habituation are also impaired by AβPP over-expression and Aβ deposition [15], [21], [22], which can seriously confound the interpretation of cross-habituation levels. We suggest the current results more accurately portray the odor discrimination ability of these mice.

Basic odor discrimination is notoriously robust in the face of severe damage to the central olfactory system, including massive lesions of the OB and other regions [46]-[49] although very fine olfactory acuity can be more sensitive to damage or circuit function disruption [44], [50]-[53]. Why then is odor perceptual impairment such an early and strong predictor of transition from mild cognitive impairment to AD [2], [9], [10], [54]? Although all aspects of olfaction can be impaired in early stages of AD or in those at risk for AD, including odor detection and discrimination [55], it is increasingly apparent that in humans, odor identification is the most strongly affected aspect of odor perception [2], [3], [16], [54], [56], [57]. For example, a meta-analysis of over 80 studies on olfaction in Parkinson's disease and AD revealed that while Parkinson's disease is more commonly associated with deficient odor detection and low level olfactory abilities, AD was more strongly associated with deficient higher cognitive olfactory abilities such as identification [3]. Primary impact on odor identification with relatively spared discrimination may suggest that early AD pathology may be influencing connectivity of the olfactory system with other regions more involved with identification, in addition to the primary olfactory system itself. In addition, AD is characterized by early disruption of modulatory systems such as the noradrenergic locus coeruleus [8] and the cholinergic basal forebrain [58]. These are two systems known to modulate odor perception and memory [53], [59], [60], and which are relatively spared in the Tg2576 mouse.

Thus, our data show that performance in a two-alternative forced choice task may not be an ideal assay of processing comparable to odor identification, even with the difficult discrimination task involving odor mixtures overlapping by 90%. Animal models of odor identification have been developed which involve cross-modal association conditioning allowing animals to identify odors through choice of specific learned somesthetic cues [61], [62]. Whether such tasks would be sensitive to AβPP over-expression and/or Aβ deposition remains to be explored. Even though basic odor discrimination relies on memory and synaptic plasticity [12], [63], odor identification may be expected to be much more heavily memory dependent as it may rely on an association between the percept and a verbal label or cross-sensory cue. Damage to the targets of the PCX in AD may contribute to such an associative impairment, for example the entorhinal or orbitofrontal cortices, two regions with strong odor processing roles [64]. The fact that the entorhinal cortex is an early target of AD related neuropathology [65] makes it an especially important region for investigation.

The present study sought to investigate the effect of AβPP-related pathology on olfactory processing in aPCX. Our results demonstrate that single-unit olfactory processing and behavior is especially robust in the face of elevating levels of Aβ accumulation through disease progression. While there were findings of baseline single-unit hyper-excitability, odor processing remained largely intact in individual neurons in aPCX. This correlated with intact odor discrimination in Tg2576. However, recent work suggests that higher order olfactory functioning may be more sensitive to pre-clinical AD and in those predisposed to AD than odor detection or discrimination [2], [3], [54]. Thus, it will be important to develop better behavioral assays for olfactory identification in animal models, as well as to explore the effects of AβPP-metabolite pathology on information transfer from the primary olfactory system to circuits involved in olfactory cognition. Olfaction remains a unique opportunity to develop early biomarkers of AD, and improve early treatments and/or preventatives.
